# Peripheral nerve injuries in children—prevalence, mechanisms and concomitant injuries: a major trauma center’s experience

**DOI:** 10.1186/s40001-023-01082-x

**Published:** 2023-03-12

**Authors:** Martin Aman, Kim S. Zimmermann, Arne H. Boecker, Mirjam Thielen, Florian Falkner, Simeon Daeschler, Annette Stolle, Ulrich Kneser, Leila Harhaus

**Affiliations:** Department of Hand-, Plastic and Reconstructive Surgery, Burn Center, BG Trauma Center Ludwigshafen, Department of Hand- and Plastic Surgery, University of Heidelberg, Ludwig-Guttmann-Str. 13, 67071 Ludwigshafen, Germany

**Keywords:** Peripheral nerve injury, Pediatric nerve injury, Trauma, Epidemiology, Reconstruction, Nerve treatment, Children, Adolescents

## Abstract

**Background:**

Peripheral nerve injuries are severe conditions with potential lifelong impairment, which is especially meaningful for the pediatric population. Knowledge on prevalence, injury mechanisms and concomitant injuries is, therefore, of utmost importance to increase clinician awareness and enable early diagnosis and treatment. As current literature on pediatric nerve lesions and concomitant injuries is scarce, we aimed to analyze all details of our patient population.

**Methods:**

A total of 110 667 patients treated at our level 1 trauma center from 2012 to 2021 were evaluated for pediatric peripheral nerve injuries, causes, concomitant injuries and assessed for lesion classification (in continuity, partial lesion, dissection) and further relevant intraoperative findings.

**Results:**

We found 5026 patients of all ages with peripheral nerve lesions, whereof 288 were pediatric, resulting in a prevalence of 5.7% of pediatric patients with nerve injuries. Mean age was 12.4 ± 4.6 years. Most common lesions were digital nerves (48.2%), followed by median (14.9%), ulnar (14.6%), radial (8.8%), peroneal nerve (5.2%) and brachial plexus injuries (2.1%). Of all pediatric nerve injuries, 3.8% were iatrogenic, only 30.2% had preserved continuity and 47.3% a concomitant vessel injury. Fractures were accompanied in 22.6%.

**Discussion:**

We observed that a large proportion of injures had complete transections, often accompanied by concomitant vessel injuries especially in distally located injuries, highlighting the importance of early surgical exploration. Radial, ulnar and lower extremity nerve injuries were often associated with fractures. Early surgical nerve repair is key to improve motor and sensory outcomes. Knowledge on mechanisms and concomitant injuries facilitates timely diagnosis and treatment, thereby potentially preventing lifelong impairment.

## Background

Peripheral nerve injuries are severe conditions often leading to lifetime impairment in case of incomplete recovery. This is especially relevant for children, who may potentially suffer for decades [1, 2].

It is widely accepted, that peripheral motor nerve lesions only have a limited time window of 18 months for sufficient muscle reinnervation, which competes directly with a regeneration rate of only approximately 1 mm per day [1]. As peripheral nerve lesions are often diagnosed with a certain delay, remaining time for successful treatment can be short [3]. To raise clinician awareness for these debilitating injuries, evidence on their prevalence, most common mechanism of injury and potential concomitant injuries are key to facilitate timely diagnosis and treatment.

However, available epidemiologic data on nerve injuries are scarce, particularly in pediatric patients. Previous studies relying on data from national health registries, often provide limited details on mechanisms or concomitant injuries [4] whereas detailed data sets are only available for small patient numbers [5]. Birch et al. [1] estimated a prevalence of 10–15% for traumatic peripheral nerve injuries among children in large trauma centers. Furthermore, there is no evidence on nerve continuity characteristics and intraoperative findings of individual nerve injuries, although this might have a high impact on clinical decision making.

We, therefore, aimed to retrospectively analyze one of the largest cohorts of pediatric nerve injuries treated in a German level one trauma center specialized in peripheral nerve injures to improve our understanding of these potentially debilitating entities in children.

## Methods

A total of 110 667 patients were identified by retrospective evaluation of all patients treated at our level 1 trauma center from January 2012 to July 2020.

Inclusion criteria: all patients with peripheral nerve lesions, including compression syndromes and iatrogenic nerve lesions were identified using the digital hospital information system and International Classification of Diseases (ICD) Classification System. All ICD codes used for evaluation are listed in Table 1. Data acquisition was performed by two independent reviewers (MA, KSZ) in a pseudonymized manner. Patients under 18 years were extracted for analysis.

**Table 1:** Summary of most common mechanisms of injury and the intraoperatively revealed nerve continuity as well as most common concomitant injury

Nerve	Main cause of injury	Intact nerve continuity	Most common concomitant injury
Digital n.	96.2% laceration	1.9%	58.2% vessel injury
Median n.	51% laceration	36.7%	38.8% tendon injury
Ulnar n.	45.8% falls	62.5%	37.5% fracture
Radial n.	34.5% laceration	62.1%	48.3% fracture
Brachial plexus	42.9% motor vehicle	58.7%	none
Sciatic n.	50% falls / 50% motor vehicle	100%	25% multiple tendons and vessels
Peroneal n.	41.2% motor vehicle	64.7%	47.1% fracture
Tibial n.	42.9% burn	57.1%	28.6% fracture

Exclusion criteria: all nerve lesions occurring as a typical concomitant injured structure (e.g. amputation) were not included as peripheral nerve lesion but rather considered with their main ICD classification. All patients treated by our specialists in various other cooperation-hospitals such as obstetric brachial plexus palsy are not included.

Local ethical board approval was obtained from the Landesärztekammer Rhineland Palatinate., Mainz; (EK Nr: 2021-16091).

An anonymized database was created using Microsoft Excel. SPSS Statistics Version 27 (IBM, USA) was used for statistical analysis.

Continuous data were reported as the mean and the mode (age, inpatient treatment, number of operations). Following the verification of normal distribution, differences in the distribution of continuous measures were assessed using the Student’s *T*-Test (age, inpatient treatment) and the distribution of categorical factors were analyzed by means of the Pearson’s *χ*^2^ Test (weekly distribution, seasonal distribution). For variables with only two values, the test for binomial distribution was used to detect significant differences (sex, affected side). Statistical significance was determined with a two-sided *p*-value < 0.05 and an alpha level of 0.05 was chosen for all tests.

Data presentation was in accordance with SAMPL guidelines and data are displayed as M (SD) = mean (standard deviation).

## Results

### Demographics

We analyzed a total of 110 667, patients whereof 5026 patients of all ages were treated for peripheral nerve lesions at our level 1 trauma center from January 2012 to July 2020. Thereof, 288 were children and young adolescents under the age of 18 whereas 4738 were adults.

This indicates a proportion of 5.7% children among all patients suffering from peripheral nerve lesions and an overall prevalence of 0.26% of children suffering from a peripheral nerve lesion among all trauma patients.

**Fig. 1:**
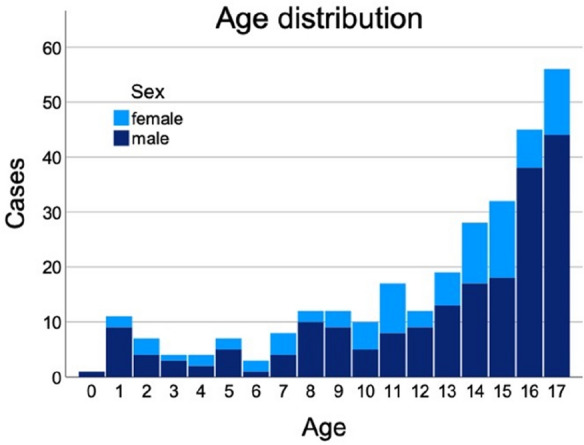
Age distribution of peripheral nerve lesions among children according to age and sex

Mean age was 12.4 (4.6) years. 200 patients (69.4%) were boys and 88 (30.6%) were girls (*p* < 0.001). Lesions occurred in 50.7% on the left and 47.6% on the right side (*p* = 0.634), with bilateral injuries occurring in only 1.7% (Fig. 1).

**Fig. 2:**
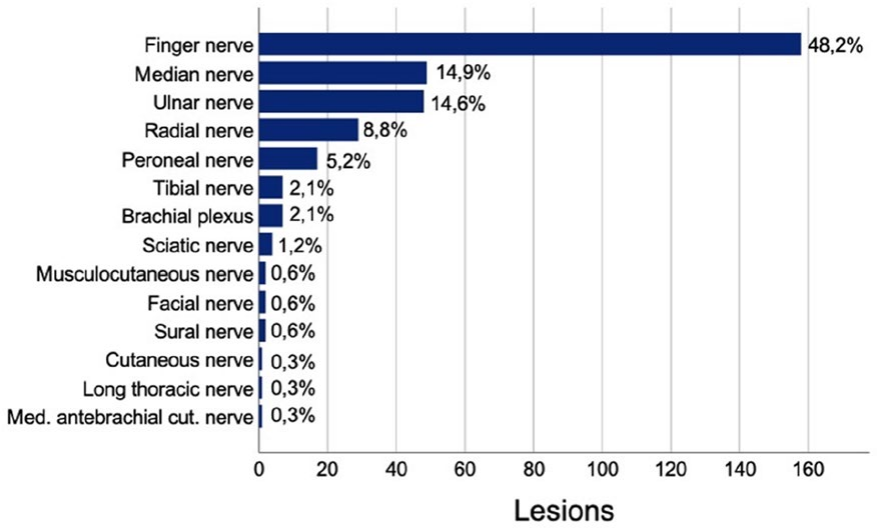
Frequency of individual nerve lesions among children

Digital nerve injuries were most common (158; 48.2%) among children followed by nerve lesions proximal to the wrist affecting the median nerve (49; 14.9%), ulnar nerve (48; 14.6%), radial nerve (29; 8.8%), and acute traumatic brachial plexus injuries (7; 2.1%), as well as peroneal nerve lesions (17; 5.2%) (Fig. 2).

The most common injury causes in pediatric patients were lacerations and cutting injuries (196; 68.1%) followed by falls (42; 14.6%) and motor vehicle accidents (16; 5.6%). Hereby, 273 cases (94.8%) had a traumatic cause, 11 (3.8%) were iatrogenic and 4 (1.4%) had a non-traumatic (idiopathic) cause.

Nerve continuity was found to be preserved in 30.2% (crush injury). Accordingly, 56.4% had a complete, 10.7% an incomplete transection and in 2.7% of lesions no information on continuity could be acquired.

In most cases (52.7%) an isolated nerve lesion was diagnosed, whereas in, 35.4% one vessel and in 11.9% at least two concomitant vessels (esp. finger nerve injuries—see finger nerve section) were affected. Overall, 22.6% of lesions were accompanied with a fracture.

In total, the children spent a mean of 6.1(0.6) nights in the hospital, but with a mode of three days.

In 98.3% of the cases, only one nerve-specific operation was performed.

Analysis for distribution of accumulations on weekdays revealed a peak of accidents on Saturday (20.1% of cases) followed by Tuesday (16.1%) showing a trend but no significant difference (p = 0.09).

### Individual nerve characteristics

#### Finger nerves

We identified 158 cases (48.2%) with lesions to finger nerves. Mean age of these patients was 11.8 (0.4) years.

**Fig. 3:**
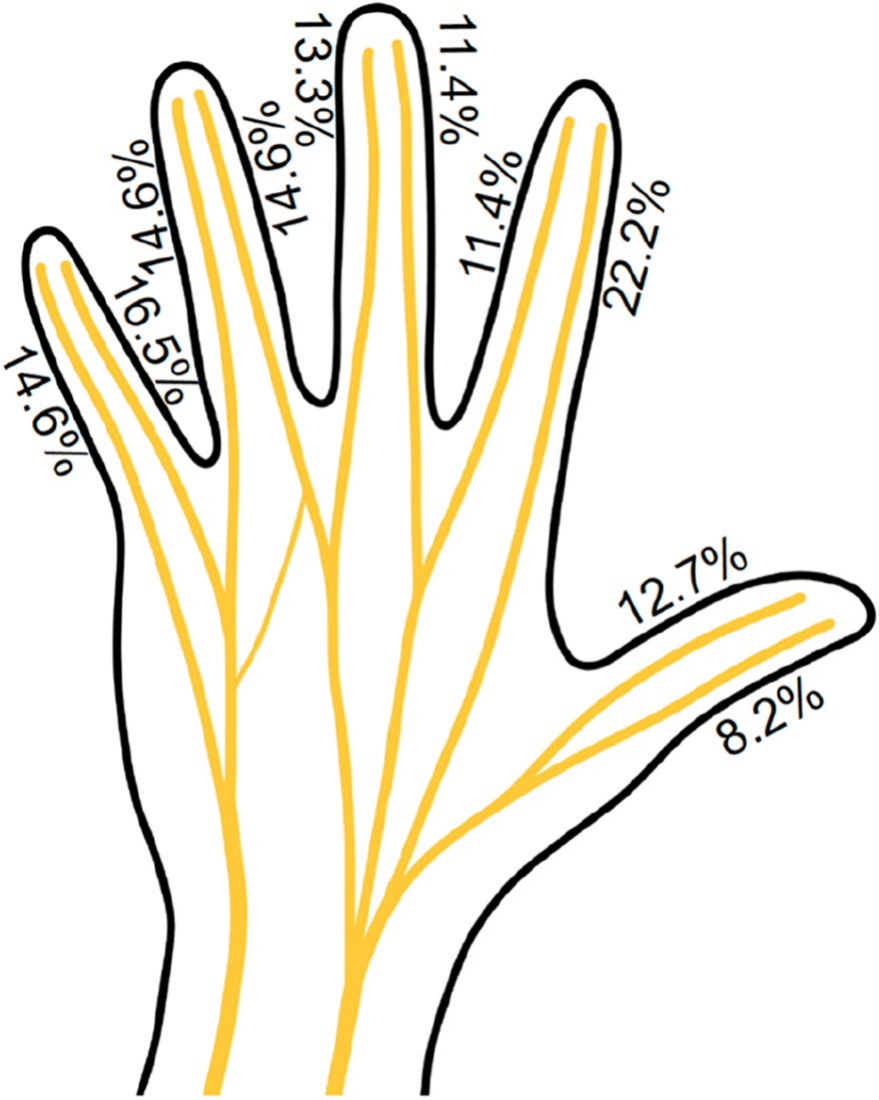
Prevalence of injured finger nerves. Shown are all 10 volar nerves of the fingers and their individual frequency of injury in isolated and combined finger nerve injuries

Main injury cause of these lesions was with 96.2% laceration injuries, in 1.9% trauma mechanism was unknown and 1.3% were due to bite injuries. Direct mechanism was in 98.1% a transection of the nerve and in 0.6% either compression, neuroma formation, or nerve irritation/elongation. Hereby, 88.6% had a complete transection of the nerve, 9.5% an incomplete transection and only 1.9% of finger nerves showed intact continuity. Analyzing concomitant injuries showed 57% singular nerve injuries without any tendon, bone or vessel injury, and in 16.5% of the lesions one and in 22% at least two tendons were injured. Furthermore, we found in 58.2% a concomitant vessel injury and in 14.9% at least two injured vessels. Only 27.8% had an isolated finger nerve injury without further vessel injury. In 9.5% of the lesions was a fracture present. Inpatient treatment showed a mean of 3.7 (0.2) nights (Fig. 3).

#### Median nerve

The median nerve proximal to the wrist was affected in 49 cases (14.8%). Main causes were lacerations (51%) followed by falls (26.5%) and 6.1% were due to motor vehicle accidents. 8.2% had non traumatic or post-traumatic lesions, such as nerve compression syndromes. Patients with median nerve injuries had a mean age of 12.6 (4.3) years.

Direct mechanism of nerve lesion was identified in 63.3% with transection of the nerve, 18.4% were revealed as a compression as well as another 18.4% with nerve irritation/ elongation. Intraoperative evaluation revealed 36.7% of the lesions with an intact nerve continuity, 32.7% with incomplete and 30.6% with a complete transection of the nerve.

38.8% of median nerve lesions had a concomitant tendon injury and 28.6% a further vessel injury. 24.5% of median nerve lesions were accompanied by a fracture.

Inpatient treatment showed a mean of 5.5 (0.9) nights.

#### Ulnar nerve

The ulnar nerve was injured in 48 cases (14.6%). Main injury causes were falls (45.8%) followed by lacerations (33.3%) motor vehicle accidents (8.3%) and in another 8.3% no trauma was found. Direct mechanism of nerve lesion intraoperatively were lacerations (37.5%) followed by nerve irritation/elongation (35.4%) and compression (27.1%). Hereby, 62.5% of nerve continuity was found intact, 31.3% of the lesions had a complete transection and 6.3% an incomplete transection. Mean age of patients with an ulnar nerve lesion was 12.2 (0.5) years.

Concomitant injuries revealed that 37.5% had a fracture, 29.2% a vessel injury and 29.2% an accompanied tendon injury. Inpatient treatment showed a mean of 6.3 (1.0) nights.

#### Radial nerve

The radial nerve was affected in 29 (8.8%) cases. Main causes of injury were lacerations cuts (34.5%), followed by falls (27.6%) and in 20.7% no trauma was found. Furthermore, 6.9% were due to motor vehicle accidents, 6.9% due to various trauma and 3.4% because of gunshot or explosion wounds. Direct mechanism was identified in 41.4% with irritation/elongation, in 47.9% with transection and 20.7% with compression. Thereof, 62.1% of nerve continuity was intact, 34.5% of radial nerves were completely transected and 3.4% incomplete transected. Mean age of the patients was 14.4 (0.4) years.

Concomitant injuries revealed that 48.3% had a fracture. 13.8% had a concomitant vessel injury and 24.1% had a further tendon injury.

Inpatient treatment showed a mean of 7.5 (1.9) nights.

#### Brachial plexus

Traumatic brachial plexus injuries (non-obstetric brachial plexus injury (OBPI)) occurred in seven cases (2.1%). Mean age was 14.9 (1.2) years. Main cause of these injuries were due to motor vehicle injuries (42.9%), traction injuries (28.6%) cuts (14.3%) and various trauma (14.3%). Intraoperative findings revealed the main mechanisms were elongation and irritation in 57.1%, 28.6% had an unclear direct cause and 14.3% had in-continuity neuroma formation. 58.7% of nerve continuity was found intact. Concomitant injuries revealed no concomitant vessel or tendon injury or fracture.

Inpatient treatment showed a mean of 8.0 (4.0) nights.

#### Sciatic nerve

Four cases of sciatic nerve lesions were found (1.2%). Mean age was 15.3 (2.1) years. Causes were falls (50%) and motor vehicle injuries (50%). Direct mechanism was found in 75% as elongation and irritation of the nerve and in 25% as a compression on the nerve. Nerve continuity was intact in 100% of the lesions. Concomitant injuries revealed in 25% of the lesions multiple tendon and vessel injuries but no fractures.

Inpatient treatment had a mean of 26.8 (15.5) nights.

#### Peroneal nerve

We identified 17 cases (5.2%) of peroneal nerve lesions. Mean age was 15.5 (0.6) years. Main causes of the peroneal nerve lesion were motor vehicle accidents (41.2%), falls (23.5%), electricity injuries (17.6%), 5.9% had a cutting injury and 11.8% had non or post-traumatic lesion due to compression. Hereby the direct mechanism was identified in 52.9% with elongation/irritation, 29.4% had an unclear cause and 11.8% a transection and a further 5.9% compression. Nerve continuity was found in 64.7% intact. Hereby 29.4% had a concomitant tendon injury and 35.3% had a vessel injury. In 47.1% of the lesions, a fracture was present. Inpatient treatment had a mean duration of 32.8 nights (6.3) nights but with a mode of five nights.

#### Tibial nerve

A tibial nerve lesion was found in seven cases (2.1%). Main cause was identified in 42.9% as electricity or burn injury, in 28.6% due to falls and 14.3% with motor vehicle injuries and another 14.3% as traction injury. Mean age of the patient was 15.7 (0.4) years. Direct mechanism was identified in 42.9% as elongation/irritation, in another 42.9% with an unclear cause and in 14.3% as (mainly post-traumatic) compression. Nerve continuity was found intact in 57.1% of the lesions. Concomitant injuries revealed in 28.6% a concomitant vessel or tendon injury. 28.6% had a fracture. Inpatient treatment had a mean duration of 42.7 (13.3) nights (Table 1).

## Discussion

Peripheral nerve lesions are devastating conditions especially for children, which require early diagnosis and treatment to minimize the rate of impaired outcome [2]. Often, concomitant injuries like fractures are initially more apparent and treated with priority [6]. Not seldom, a phase of cast-immobilization follows, which may further conceal potential motor and sensory deficits resulting from concomitant nerve injury [7]. This is a critical situation especially in pediatric population since children may not always be able to notice and describe nerve related symptoms clearly. Valuable time for the best possible treatment is lost [3, 8].

This explains the necessity to focus especially on these injuries to increase the awareness for diagnosing such injuries. Current literature hereby lacks detailed data on peripheral nerve lesions in children. Therefore, we aimed to analyze our patients who are presented with a peripheral nerve lesion at our major trauma center.

We identified a prevalence of children of 0.26% among all trauma patients. For patients with nerve lesions, we found a prevalence of 5.7% of children among our patients. This low number is in accordance with findings from Missios et al.[4] who found 0.56% in a large national health registry. Compared to this study [4] with an U.S collective and motor vehicle injuries as main cause for trauma, we found the vast majority of peripheral nerve injuries being caused by cuts or lacerations with mainly injuries to the finger nerves in our population. A direct comparison to this study seems impossible as they did not show terminal branches such as finger nerves.

Beside finger nerve injuries, the median and ulnar nerves were the most frequently injured nerves in our collective. This might be due to anatomical proximity to structures frequently injured in children such as distal humerus and elbow as well as radial fractures [8]. Hereby radial nerve injuries were in almost half of the cases accompanied by a humerus fracture and the ulnar nerve in one-third of the cases.

We only identified 62% of the radial nerve injuries being in-continuity which is in contrast to findings of other author’ findings of 90% [9]. This has a high clinical impact when considering early exploration in doubt of recovery or unclear MRI findings due to associated humerus plates [10].

Numbers in lower extremity were even higher with 64.7% of concomitant tibia / fibula fractures in patients with peroneal nerve lesions and 57.1% in tibial nerve lesions, respectively. This indicates the necessity of detailed examination of children with fractures.

**Fig. 4:**
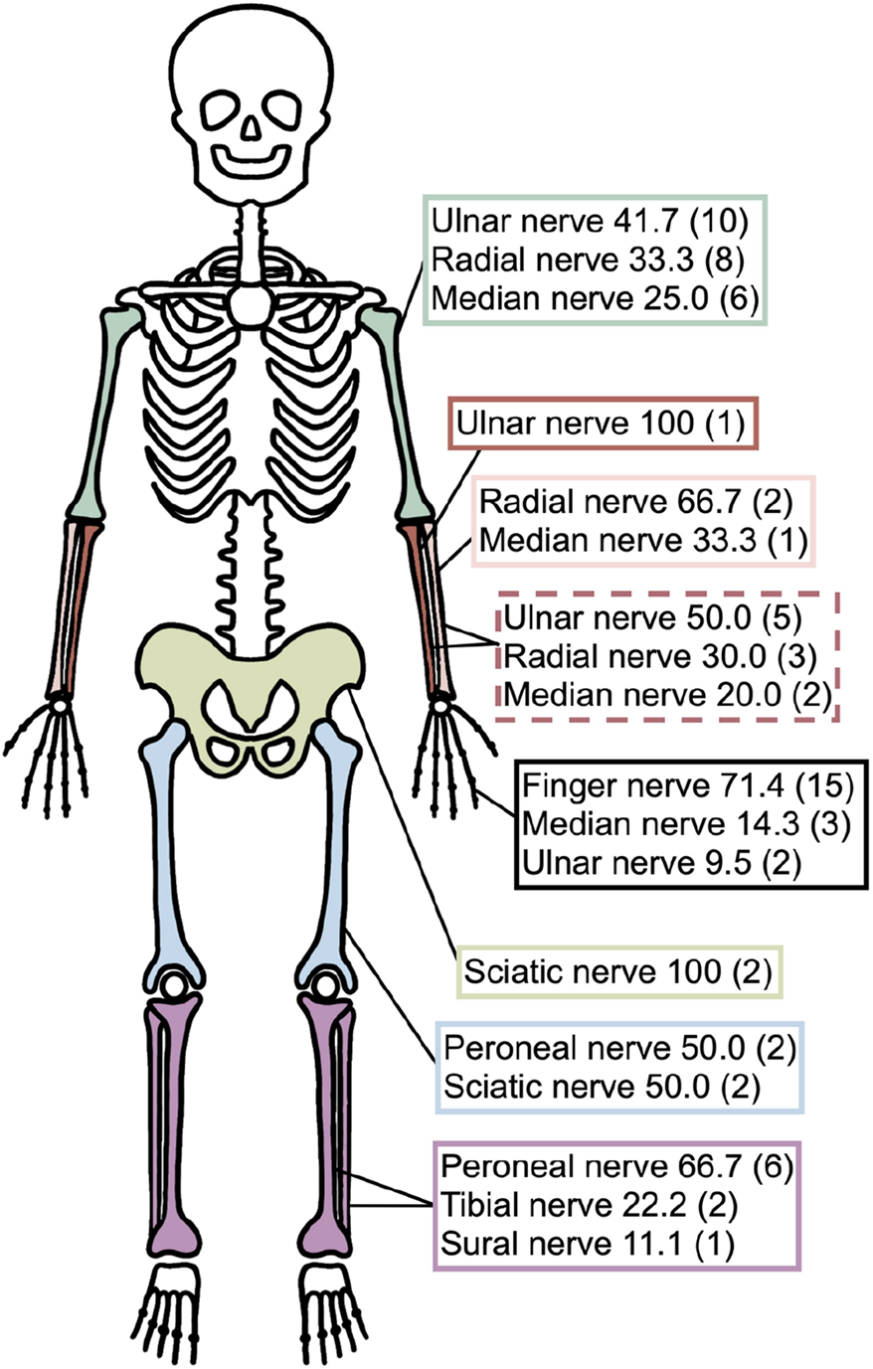
Concomitant fractures in peripheral nerve lesions. Displayed are colorized bones with the concurring nerve lesion in % and (absolute numbers). Dotted box represents combined ulnar and radial fracture. For example, if a humeral fracture and a peripheral nerve injury were found, in 41.7% the ulnar nerve was affected and the radial nerve in 33.3% followed by median nerve in 25% of cases

Usually injuries to the lower leg require higher force of impact for fractures, accompanied with higher concomitant injuries, longer inpatient treatment and worse outcome [6].This might also explain the significantly longer inpatient stay and a higher number of necessary operations of lower extremity peripheral nerve lesions compared to lesions in the upper extremity (Fig. 4).

Nerve continuity was intact in only 30.2% of the cases. This leaves more than two-thirds of the children with a potential nerve lesion with at least an incomplete or, even worse, with a complete transection of the nerve (56.4%). Hereby, we strongly recommend surgical exploration especially in distal injuries. Our analysis shows that 88.6% of finger nerve injuries had a complete transection of the nerve. Furthermore, in 58.2% we identified an accompanied vessel injury intraoperatively.

With only limited reliability of clinical examination in children, lacerations of the hand should be operatively explored for nerve and vessel injuries rather than treated by simple wound closure alone.

Without early surgical treatment this may lead to lifelong impairment [1, 2, 4]. Due to the young age, this results not only in high individual burden for patients and their families due to dependency in daily life activities and psychological burden but furthermore to high socioeconomic costs for society. We could demonstrate high costs for injuries and compensation for adult patients suffering from peripheral nerve injuries of more than 16 000 euros per year in a previous work [11]. In young patients, this socioeconomic burden may be further amplified.

Luckily, it is generally accepted that children have a higher recovery rate for peripheral nerve injuries compared to adults when treated adequately. For example, Lohmeyer et al. [12] found a superior recovery of sensation after finger nerve injury in children compared to even young adults and further declining with decades of patient age. This is also supported by experimental data found in animal trials [13].

This may be due to shorter regeneration distances, increased capacity for central and peripheral plasticity and a potentially a higher rate of axonal regeneration [14, 15].

We observed only 3.8% of all included pediatric nerve lesions to be iatrogenic. In the current literature, there is only very limited data on overall numbers of iatrogenic nerve lesions in children. Some authors suggest up to 35.1% for supracondylar humerus fracture operations, which we could not verify in our population [16]. Again, as examination of pediatric patients is often limited and cast immobilization might conceal functional impairment, in doubt detailed clinical, neurological and radiological examination should be performed.

An interesting finding was the high prevalence of lesions due to burns and electricity injuries in the peroneal and tibial nerve. This has been previously described by Marquez et al. [17]. Latest research in animal models and clinical examinations suggest several pathomechanisms such as Wallerian degeneration due to the burn itself as well as a high number of cytokines in burn patients affecting conduction velocity. Higher compartment pressure and compartment syndromes may play a major role as well [18] .

Limitations

This retrospective data collection is limited to the regular way of daily clinical routine documentation. Therefore, detailed outcome after injuries is documented in a heterogenous fashion which does not allow reliable data acquisition on outcome after these lesions. That’s why we excluded outcome reports from our analysis. Further clinical studies will be necessary to evaluate long-term outcomes. Also, this data represents patients treated at our special facility. Our nerve center is one of the largest in Germany with a large catchment area without further surrounding nerve lesion-treating facilities. All patients treated by our specialists in various other cooperation-hospitals such as obstetric brachial plexus palsy are, therefore, not included.

## Conclusion

This analysis of peripheral nerve lesions in children and young adolescents treated at a German level one trauma center demonstrates a prevalence of 5.7% among patients of all ages with a peripheral nerve lesion and a total prevalence of 0.26% among all trauma patients.

Hereby, lacerations to the finger nerves with complete transection of the nerve were the most common injury. More proximal lesions have a higher chance of being accompanied by a fracture compared to distal lesions. The ulnar and radial nerves seem to be very prone to injury especially in distal humerus fractures. Also, lower extremity fractures should be examined carefully for nerve injury. Given the high proportion of discontinuity nerve lesions requiring surgical repair, referring children with suspected nerve injuries to specialized centers may facilitate early diagnosis, timely treatment and adequate rehabilitation and thereby may prevent lifelong impairments in children suffering from nerve injuries.

## Data Availability

The datasets used and/or analysed during the current study are available from the corresponding author on reasonable request.
